# Effect of the *ALDH2* Variant on the Prevalence of Atrial Fibrillation in Habitual Drinkers

**DOI:** 10.1016/j.jacasi.2021.10.009

**Published:** 2022-01-18

**Authors:** Takayoshi Yamashita, Yuichiro Arima, Tadashi Hoshiyama, Noriaki Tabata, Daisuke Sueta, Yusei Kawahara, Miwa Ito, Hisanori Kanazawa, Masanobu Ishii, Kenshi Yamanaga, Shinsuke Hanatani, Seiji Takashio, Satoshi Araki, Satoru Suzuki, Eiichiro Yamamoto, Koichi Kaikita, Kentaro Oniki, Junji Saruwatari, Kenichi Matsushita, Kenichi Tsujita

**Affiliations:** a Department of Cardiovascular Medicine, Graduate School of Medical Sciences, Kumamoto University, Kumamoto, Japan; b Division of Pharmacology and Therapeutics, Graduate School of Pharmaceutical Sciences, Kumamoto University, Kumamoto, Japan

**Keywords:** alcohol, aldehyde dehydrogenase, atrial fibrillation, East Asians, AF, atrial fibrillation, *ALDH2*, aldehyde dehydrogenase 2, CI, confidence interval, ECG, electrocardiogram, OR, odds ratio

## Abstract

**Background:**

Alcohol—a risk factor for atrial fibrillation (AF)—is metabolized by aldehyde dehydrogenase 2 (*ALDH2*). Dysfunctional alleles of *ALDH2* (*ALDH2*-deficient variants) are prevalent among East Asians.

**Objectives:**

Because the prevalence of AF is estimated to be high in *ALDH2*-deficient variant carriers, we investigated the correlation between AF and *ALDH2*-deficient variant carriers, including the association with habitual alcohol consumption.

**Methods:**

A total of 656 consecutive patients were included in this investigation. *ALDH2* genotypes were divided into *ALDH2* homozygous wild-type (*∗1*/*∗1*), *ALDH2* heterozygous-deficient allele (*∗1*/*∗2*), and *ALDH2* homozygous-deficient allele (*∗2*/*∗2*). Multivariate analyses were applied to determine the correlation between *ALDH2* genotype and AF.

**Results:**

*ALDH2∗1/∗2* and *ALDH2∗2/∗2* carriers who were *ALDH2*-deficient variant carriers comprised 199 (30.3%) and 27 (4.1%) patients, respectively. Among these patients, the proportions of habitual alcohol consumption were 26.1% and 0%, respectively. Multivariate analysis revealed that *ALDH2∗1/∗2* itself was not a risk factor for AF (odds ratio [OR]: 1.28; *P* = 0.21). However, habitual alcohol consumption in *ALDH2∗1/∗2* carriers was an independent risk factor of AF (OR: 4.13; *P* = 0.001). Contrary to expectations, *ALDH2∗2/∗2* itself had a lower incidence of AF among other risk factors (OR: 0.37; *P* = 0.03).

**Conclusions:**

Although the *ALDH2∗1/∗2* itself was not associated with AF, *ALDH2∗1/∗2* carriers with habitual alcohol consumption could experience AF because of slow alcohol metabolism. In contrast, *ALDH2∗2/∗2* itself had a lower incidence of AF. This might be related to the absence to habitual alcohol consumption in *ALDH2∗2/∗2* carriers because of the negligible activity of *ALDH2.* Thus, abstaining from alcohol consumption might prevent the development of AF in patients who are *ALDH2∗1/∗2* carriers.

Atrial fibrillation (AF) is the most common form of arrhythmia detected in clinical practice.^1^ AF increases the risk of stroke, cardiovascular disease, and all-cause mortality.^2^ Although it has been shown that a hyperadrenergic state and impairment of vagal tone are closely associated with AF,^3^^,^^4^ the precise mechanisms leading to AF remain unclear. Older age, male sex, hypertension, obesity, and diabetes mellitus have been shown to be risk factors for AF.^5^ Alcohol consumption, even at moderate levels, is also associated with this arrhythmia.^6^ Some reports have indicated that alcohol progresses atrial electrical remodeling and triggers AF.^7^ Another study showed that prohibition of alcohol consumption reduces AF attacks.^8^

Alcohol is metabolized in 2 steps. First, it is metabolized to acetaldehyde by an alcohol dehydrogenase subunit, and second, the acetaldehyde is metabolized to acetic acid by aldehyde dehydrogenase 2 (*ALDH2*).^6^ Dysfunctional alleles of the *ALDH2* gene are prevalent among East Asian populations.^9^ *ALDH2* genotypes consist of *ALDH2* homozygous wild-type (*∗1*/*∗1*), *ALDH2* heterozygous-deficient allele (*∗1*/*∗2*), and *ALDH2* homozygous-deficient allele (*∗2*/*∗2*). The carriers of *ALDH2∗1*/*∗2* and *ALDH2∗2*/*∗2* are *ALDH2*-deficient variant carriers. Compared with the *ALDH2* wild-type, *ALDH2∗1*/*∗2* and *ALDH2∗2*/*∗2* represent reduced and negligible activity of *ALDH2*, respectively.^9^ These findings imply that increased prevalence of AF in patients with *ALDH2∗1∗2* who habitually consume alcohol is caused by the low metabolic activity and accumulation of toxic acetaldehyde. In contrast, there is decreased prevalence of AF in patients with *ALDH2∗2/∗2*, because these patients are not able to consume alcohol because of the negligible activity of *ALDH2*. In this study, we investigated the relationship between *ALDH2* genotypes and the prevalence of AF in association with habitual alcohol consumption.

## Methods

### Patient population and data collection

Between January 2010 and June 2019, 10,603 patients were admitted to the Cardiovascular Center of Kumamoto University. Among these, 656 patients were enrolled in our study retrospectively. The purpose of admission was catheter ablation for AF in 385 patients, catheter ablation for other arrhythmias in 196 patients, investigation of aortic disease in 49 patients, and coronary angiography in 26 patients. The absence of coronary artery disease, moderate or severe valve disease, cardiomyopathy, and hyperthyroidism was confirmed in all patients. The genomic information of each patient was preserved at the time of blood sampling and obtained from our institution’s biobank. This study complied with the Declaration of Helsinki regarding investigation in humans and was approved by the Human Research Committee of Kumamoto University. Written informed consent was obtained from each patient.

The data collected from the study population, including vital signs, internal medicine history, and history and preference of alcohol, were obtained from the patient’s medical records.

In 385 patients who underwent catheter ablation for AF, AF was confirmed using surface electrocardiography (ECG) or Holter ECG. In the other patients, new-onset AF was confirmed using surface ECG and by monitoring the ECG during admission. Previously documented AF was confirmed using surface ECG or Holter ECG performed by their primary care doctor. AF was defined by the absence of P waves, presence of fibrillatory waves, and an irregular ventricular rate in patients without conduction disorders that lasted >30 s. Following this, AF was diagnosed by ≥2 cardiologists.

### *ALDH2* genotype and the allele ratio

Genomic DNA used for genotyping of *ALDH2* was extracted from whole blood using a DNA purification kit (Flex Gene DNA kit, Qiagen). The *ALDH2* rs671 (Glu504Lys; *ALDH2∗2*) genotypes were determined by performing a real-time TaqMan allelic discrimination assay (Step One Plus Real-Time PCR system version 2.1, Applied Biosystems) according to the manufacturer’s protocols (assay no. C_11703892_10). The allele ratio was analyzed using this method.

### Variables

The following variables were analyzed: age, sex, hypertension, diabetes mellitus, obesity, alcohol consumption habit, and *ALDH2* genotypes. We defined older age as older than 60 years, because the prevalence of AF increases in individuals above this age.^10^ Diabetes mellitus was defined based on the casual plasma glucose concentration of >200 mg/dl, fasting plasma glucose concentration of >126 mg/dl, 2-h plasma glucose concentration of >200 mg/dl in a 75-g oral glucose tolerance test, and administration of diabetes mellitus medication. Hypertension was defined in patients with blood pressure of >140/90 mm Hg or administration of antihypertensive medication. Obesity was categorized in subjects with a body mass index of >25 kg/m^2^. Habitual alcohol consumption was defined as ≥3 drinking opportunities per week, according to the Ministry of Health, Labour and Welfare in Japan.

### Data analysis

To determine the correlation between the *ALDH2∗2* allele and AF, univariate and multivariate analyses were performed to identify factors associated with AF.

In addition, we evaluated the relationship between AF and habitual alcohol consumption, based not only on categorical diagnosis, but also with regard to total alcohol consumption in relation to the *ALDH2* genotype. Average alcohol intake volume was obtained from patients’ self-report. The volume was represented in standard drinks per week. One standard drink was defined as 12-g alcohol. According to this definition, alcohol intake volume was divided into the following groups: nondrinkers: 0-1 drinks/week; mild drinkers: 2-7 drinks/week; moderate drinkers: 8-21 drinks/week; and heavy drinkers: ≥22 drinks/week.^11^

### Statistical analysis

All continuous data showed a normal distribution according to the Shapiro-Wilk test; continuous data were expressed as mean ± SD. One-way analysis of variance with Tukey’s test was used for multiple comparisons of data. Continuous data that showed a non-normal distribution using the Shapiro-Wilk test were expressed as the median (interquartile range). The Kruskal-Wallis with Steel-Dwass test was used for multiple comparisons of the data. Categorical data were presented as numbers or percentages. Differences between multiple groups were tested using Fisher’s exact test with Bonferroni’s correction. Multiple logistic regression analysis was performed to determine the risk factors for AF. Propensity-score matching and inversed probability treatment weighting analyses were performed to balance the bias of background. Cox regression analysis was performed to determine the risk factors for new-onset AF. A *P* value <0.05 was considered statistically significant. Statistical analyses were performed using SPSS version 25 (IBM), except for propensity-score matching, which was performed using R (The R Foundation for Statistical Computing).

## Results

### Patient characteristics

Among the 656 patients, the distribution of the genotypes *ALDH2∗1/∗1* (*ALDH2* wild-type) and *ALDH2*-deficient variant carriers, which included *ALDH2∗1/∗2* and *ALDH2∗2/∗2* carriers, were 430, 199, and 27 patients, respectively. This proportion of *ADLH2* genotypes was similar to the reports in Japanese and East Asian populations.^9^^,^^12^

Table 1 lists patient characteristics. There was no significant difference among *ALDH2* wild-type carriers and *ALDH2∗1∗2* and *ALDH2∗2/∗2* allele carriers except for left atrial dimensions, drinking habits, and AF. The differences of left atrial dimensions between each genotype were not significantly different. However, prevalence of habitual alcohol consumption in *ALDH2∗2/∗2* carriers was significantly less than that of *ALDH2* wild-type and *ALDH2∗1/∗2* carriers (0 of 27, 0% vs 231 of 430, 53.7%; *P* < 0.001; 0 of 27, 0% vs 51 of 199, 25.6%; *P* = 0.003). In addition, prevalence of habitual alcohol consumption in *ALDH2∗1/∗2* carriers was significantly less than that of *ALDH2* wild-type carriers (51 of 199, 25.6% vs 231 of 430, 53.7%; *P* < 0.001). Prevalence of *ALDH2∗2/∗2* carriers’ history of AF was also significantly less than those of *ALDH2* wild-type and *ALDH2∗1/∗2* carriers (9 of 27, 33.3% vs 261 of 430, 60.7%; *P* = 0.02; 9 of 27, 33.3% vs 122 of 199, 61.3%; *P* = 0.02). However, there was no significant difference between *ALDH2* wild-type and *ALDH2∗1/∗2* carriers.

**Table 1:** Patient Characteristics

	All (N = 656)	*ALDH2* Wild (n = 430)	*ALDH2∗1/∗2* (n = 199)	*ALDH2∗2/∗2* (n = 27)	*P* Value
Age, y	64 (55-71)	63 (53-71)	65 (58-71)	68 (57-72)	0.42
Male	403 (61.4)	273 (63.5)	112 (56.3)	18 (66.7)	0.20
BMI, kg/m^2^	23.2 (20.9-25.6)	23.2 (21.1-25.7)	23.2 (20.8-25.4)	23.1 (21.8-26.2)	0.65
BNP, pg/mL	28.3 (12.9-66.4)	28.5 (13.6-65.9)	28.0 (12.6-69.1)	17.3 (10.4-50.3)	0.60
EF, %	63.3 (60.0-66.2)	63.5 (60.0-66.7)	62.9 (59.8-65.6)	64.5 (61.1-67.4)	0.13
LADs, mm	35.9 ± 6.3	36.3 ± 6.3	35.2 ± 6.3	34.3 ± 6.5	0.03
eGFR, mL/min/1.73 m^2^	72.0 (62.0-85.0)	72.0 (63.0-85.0)	70.0 (60.0-84.0)	65.0 (56.5-81.0)	0.09
Atrial fibrillation	392 (59.8)	261 (60.7)	122 (61.3)	9 (33.3)	0.02
Hypertension	343 (52.2)	236 (54.9)	96 (48.2)	11 (40.7)	0.14
Diabetes mellitus	92 (14.0)	54 (12.3)	35 (17.6)	3 (11.1)	0.23
Drinking habit	282 (43.0)	231 (53.7)	51 (25.6)	0 (0)	<0.001
Current smoking	129 (19.7)	88 (20.4)	36 (18.0)	5 (18.5)	0.78

Values are median (interquartile range), n (%), or mean ± SD.

*ALDH2* = aldehyde dehydrogenase; BMI = body mass index; BNP = brain natriuretic peptide; EF = ejection fraction; eGFR = estimated glomerular filtration rate; LAD = left atrial dimension.

Table 2 shows the relationship between *ALDH2* genotypes and type of AF. As shown, the proportion of paroxysmal, persistent, and longstanding persistent AF was not significantly different between each *ALDH2* genotype.

**Table 2:** Relationship Proportion of AF Types of Each *ALDH2* Genotypes

	*ALDH2* Wild (n = 430)	*ALDH2∗1/∗2* (n = 199)	*ALDH2∗2/∗2* (n = 27)	*P* Value
Paroxysmal AF	197 (45.8)	93 (46.7)	8 (29.6)	0.24
Persistent AF	62 (14.4)	29 (14.6)	1 (3.7)	0.32
Longstanding persistent AF	2 (0.5)	0 (0.0)	0 (0.0)	1.00

Values are n (%).

AF = atrial fibrillation.

### Relationship between habitual alcohol consumption and prevalence of AF

Table 3 shows the relationship between AF and examined variables. Older age, hypertension, being male, and a pattern of habitual alcohol consumption were significantly correlated with AF. In addition, habitual alcohol consumption also exhibited a high odds ratio (OR) based on univariate (OR: 2.31; *P* < 0.001) and multivariate (OR: 1.75; *P* < 0.001) analyses.

**Table 3:** Relationship Between AF and Variables

	Univariate Analysis			Multivariate Analysis		
	OR	95% CI	*P* Value	OR	95% CI	*P* Value
Age >60 y	2.49	1.79-3.46	<0.001	2.60	1.78-3.80	<0.001
Hypertension	2.34	1.70-3.22	<0.001	1.68	1.17-2.41	0.005
Obesity	1.11	0.79-1.56	0.55	1.02	0.70-1.50	0.91
Diabetes mellitus	0.92	0.58-1.41	0.65	0.63	0.38-1.02	0.06
Male	2.12	1.54-2.93	<0.001	2.03	1.38-2.98	<0.001
Alcohol	2.31	1.67-3.21	<0.001	1.75	1.18-2.61	0.005
*ALDH2* genotype						
*ALDH2* wild		Reference			Reference	
*ALDH2∗1/∗2*	1.03	0.73-1.45	0.88	1.28	0.87-1.89	0.21
*ALDH2∗2/∗2*	0.32	0.14-0.74	0.007	0.37	0.15-0.91	0.03

CI = confidence interval; OR = odds ratio; other abbreviations as in Tables 1 and 2.

With respect to the relationship between *ADLH2* genotypes and AF history, *ALDH2∗1/∗2* carriers were not significantly correlated with AF on univariate (OR: 1.03; *P* = 0.88) and multivariate analyses (OR: 1.28; *P* = 0.21). Rather, contrary to expectations, *ALDH2∗2/∗2* carriers had a lower incidence of AF on univariate (OR: 0.32; *P* = 0.007) and multivariate analyses (OR: 0.37; *P* = 0.03).

### Relationship between habitual alcohol consumption and AF prevalence among *ALDH2* genotypes

However, the relationship between AF and *ALDH2* genotypes in regard to habitual alcohol consumption was lacking in this analysis. Therefore, variables in *ALDH2* genotypes and habitual alcohol consumption were divided into 5 categories to understand the importance of *ALDH2* genotypes and habitual alcohol consumption for the risk of developing AF. These categories were *ALDH2* wild-type carriers who did not consume alcohol habitually, *ALDH2* wild-type carriers who consumed alcohol habitually, *ALDH2∗1/∗2* allele carriers who did not consume alcohol habitually, *ALDH2∗1/∗2* allele carriers who consumed alcohol habitually, and *ALDH2∗2/∗2* allele carriers (because of the absence of habitual alcohol consumption in *ALDH2∗2/∗2* allele carriers). Multivariate analysis was performed as the reference for *ALDH2* wild-type carriers who did not consume alcohol habitually by including other variables. Table 4 shows the relationship between AF and the variables studied. This multivariate analysis was adjusted for covariates such as age, hypertension, obesity, diabetes mellitus, and being male. As shown in Table 4, age older than 60 years (OR: 2.71; *P* < 0.001), hypertension (OR: 1.64; *P* = 0.007), being male (OR: 1.86; *P* < 0.001), *ALDH2* wild-type carriers with habitual alcohol consumption (OR: 1.64; *P* = 0.037), and *ALDH2∗1/∗2* allele carriers with habitual alcohol consumption (OR: 4.13; *P* = 0.001) positively correlated with the risk of AF. Particularly, *ALDH2∗1/∗2* allele carriers who consumed alcohol habitually showed the highest OR among these variables. In addition, *ALDH2∗2/∗2* allele carriers had a lower incidence of AF (OR: 0.35; *P* = 0.02).

**Table 4:** Relationship Between AF and Variables Using Multivariate Analysis

	Habitual Alcohol Consumption	OR	95% CI	*P* Value
Age >60 y		2.71	1.84-3.98	<0.001
Hypertension		1.64	1.14-2.36	0.007
Obesity		1.03	0.70-1.51	0.88
Diabetes mellitus		0.61	0.37-1.00	0.05
Male		1.96	1.33-2.88	<0.001
*ALDH2* genotype				
ALDH2 wild	No		Reference	
ALDH2 wild	Yes	1.60	1.03-2.50	0.037
ALDH2∗1/∗2	No	1.06	0.68-1.68	0.800
ALDH2∗1/∗2	Yes	4.13	1.76-9.71	0.001
ALDH2∗2/∗2	No	0.35	0.14-0.87	0.020

Abbreviations as in Tables 1 to 3.

The previously mentioned results were based on the data of all included patients. Therefore, we performed these analyses between *ALDH2* wild-type and *ALDH2∗1/∗2* carriers using propensity-score matching analysis and inversed probability treatment weighting analyses. In this analysis, AF history and habitual alcohol consumption were included in the propensity score model as covariates. The values changed following propensity-score matching, but the overall meaning and significant difference remained the same (Supplemental Tables 1-4).

### Correlation between volume of alcohol intake and prevalence of AF among *ALDH2* wild-type and *ALDH2∗1∗2* allele carriers

Table 5 shows the relationship between AF and the alcohol intake volume categories in *ALDH2* wild-type and *ALDH2∗1/∗2* allele carriers. Moderate and heavy drinkers with the *ALDH2* wild-type allele (OR: 1.79; 95% confidence interval [CI]: 1.12-2.85; *P* = 0.01; OR: 3.07; 95% CI: 1.77-5.34; *P* < 0.001) and moderate drinkers with the *ALDH2∗1/∗2* allele (OR: 5.07; 95% CI: 2.03-12.70; *P* < 0.001) were positively correlated with AF risk. *ALDH2∗1∗2* allele carriers with moderate alcohol consumption showed the highest OR. ORs of heavy drinkers in *ALDH2∗1/∗2* allele carriers was not obtained because the proportion of AF was 100%.

**Table 5:** Relationship Between AF and Alcohol Consumption Volume in Each Genotype

	n	AF Patients	OR	95% CI	*P* Value
*ALDH2* wild-type	430	261 (60.7)			
Nondrinkers	199	102 (51.2)		Reference	
Mild drinkers	17	9 (52.9)	1.07	0.40-2.89	0.89
Moderate drinkers	121	79 (65.3)	1.79	1.12-2.85	0.01
Heavy drinkers	93	71 (76.3)	3.07	1.77-5.34	<0.001
*ALDH2∗1∗2* allele	199	122 (61.3)			
Nondrinkers	148	80 (54.1)		Reference	
Mild drinkers	7	4 (57.1)	1.27	0.28-5.81	0.76
Moderate drinkers	38	32 (84.2)	5.07	2.03-12.70	<0.001
Heavy drinkers	6	6 (100)	NA	NA	NA

Values are n (%) unless otherwise indicated.

Abbreviations as in Tables 1, 2, and 3.

### Follow-up after discharge and new-onset AF in patients without a history of AF

AF history was not observed at admission in 264 patients in the present study. Among these patients, 174 patients were followed up at our hospital. However, the follow-up was short, being only 10 months (range: 2.0-25.3 months). The number of patients who were *ALDH2* wild-type carriers, *ALDH2∗1/∗2* carriers, and *ALDH2∗2/∗2* carriers were 111, 51, and 12, respectively. During follow-up, new-onset AF was observed in only 6 patients (in each genotype, new-onset AF was observed in 2 patients). Cox regression analysis did not show the correlation between *ALDH2* genotypes and new-onset AF (Supplemental Table 4). In addition, *ALDH2* genotypes with habitual alcohol consumption also showed an absence of correlation (Supplemental Tables 5 and 6).

### Power analysis with respect to *ALDH2/∗2/∗2* CARRIERS

There were only a few *ALDH2∗2/∗2* carriers (n = 27); therefore, we conducted a power analysis with 27 *ALDH2∗2/∗2* carriers and 430 *ALDH2* wild-type carriers as control subjects. In this present study, proportion of *ALDH2* wild-type carriers with a history of AF was 0.607, and the relative risk of AF in *ALDH2∗2/∗2* carriers was 0.32. Under the type I error probability associated with this test of this null hypothesis of 0.05, the calculated power was 0.994. We used Fisher’s exact test to evaluate this null hypothesis. Therefore, we analyzed the data incorporating *ALDH2∗2/∗2* carriers as well.

## Discussion

### Main findings

In this present study, we found 3 main findings. First, the *ALDH2∗1/∗2* allele itself, which leads to the slow metabolism of alcohol, was not a risk factor for AF. However, the *ALDH2∗1/∗2* allele carriers with habitual alcohol consumption had the strongest risk factor for AF compared with older age, hypertension, and male sex. Second, with increased alcohol consumption volume, the OR of AF risk increases in both *ALDH2* wild-type and *ALDH2∗1/∗2* allele carriers. This phenomenon appears prominently in *ALDH2∗1/∗2* allele carriers. Third, the *ALDH2∗2/∗2* allele itself, which negligibly metabolizes alcohol, correlated with lower incidence of AF.

### Habitual alcohol consumption and AF

In this study, habitual alcohol consumption was one of the risk factors for AF according to the multivariate analysis.

A mechanism of developing AF based on habitual alcohol consumption may consist of various factors. First, alcohol has 2-sided effect in the atrium associated with AF, including autonomic modulation, represented by increased β-receptor density,^3^ and vagal inhibition, which leads to shortened atrial refractoriness.^4^ This is followed by atrial electrical remodeling, which is represented by lower atrial voltage and conduction slowing.^13^ These effects lead to shortened atrial and pulmonary vein action potentials, a shortened atrial effective refractory period, and slow intra-atrial and interatrial conduction. Second, it has been shown that habitual alcohol consumption and sleep apnea syndrome have a close relationship.^14^ Alcohol consumption can cause upper airway obstruction that leads to sleep apnea syndrome, and sleep apnea syndrome has been connected with developing AF because of hypoxia, hypercapnia, negative intrathoracic pressure generation, and alteration of autonomic nervous activity.^7^^,^^15^ As such, substrates of AF may be produced (7); therefore, habitual alcohol consumption is a risk factor for AF.^6^^,^^7^ These findings are supported by the positive correlation between alcohol consumption and AF observed in this study.

### Prevalence of *ALDH2* variant carriers

In this study, the distribution of the genotypes of *ALDH2∗1*/*∗1* (*ALDH2* wild-type) and *ALDH2*-deficient variant carriers, which included *ALDH2∗1*/*∗2* and *ALDH2∗2*/*∗2* carriers, was 430, 199, and 27 patients, respectively, among all 656 patients. Although the worldwide prevalence of *ALDH2∗2* allele carriers is rather small,^16^ patients who were enrolled in this present study were all Japanese. In addition, the proportion of *ALDH2∗1/∗2* and *ALDH2∗2/∗2* carriers was similar to past reports in Japanese and East Asian populations.^9^^,^^12^

### Effect of *ALDH2*-deficient variant allele on alcohol metabolism

The *ALDH2*-deficient variant allele has reduced enzyme activity.^12^^,^^17^ Therefore, low *ALDH2* activity leads to accumulation of toxic acetaldehyde in *ALDH2*-deficient variant carriers, causing an alcohol flushing syndrome in *ALDH2*-deficient variant allele carriers after alcohol consumption.^18^ In addition, some reports have suggested that the *ALDH2*-deficient variant allele contributes to cardiovascular disease, diabetes, stroke, and cancer.^19^

### Interaction between *ALDH2*-deficient variant allele carriers with habitual alcohol consumption and AF

In this study, we found that the *ALDH2∗1/∗2* allele itself was not a risk factor for AF. Consistent with the data in this study, Nakano et al^20^ reported that the *ALDH2-*deficient variant allele itself was negatively associated with AF. However, the study did not focus on habitual alcohol consumption in *ALDH2* genotypes. Being an *ALDH2∗1/∗2* allele carrier with habitual alcohol consumption was the strongest risk factor for AF in the multivariate analysis in this present study. In addition, increased alcohol consumption volume, increased the OR of AF risk, especially in *ALDH2∗1/∗2* allele carriers. It was shown that alcohol induced autonomic effects and atrial electrical remodeling, both of which are associated with the generation of the AF substrate.^7^ Following the generation of the AF substrate, atrial contraction, including triggered activity, could cause AF. In that state, accumulation of toxic acetaldehyde in *ALDH2*-deficient variant carriers could play an important role for developing AF. It was shown that overdrive pacing fails to induce triggered activity in the presence of ethanol. However, triggered activity was induced by overdrive pacing in the presence of acetaldehyde.^21^

With respect to *ALDH2∗2/∗2* allele carriers, symptoms of the alcohol flushing syndrome could be more severe because of the negligible activity of *ALDH2* compared with that of *ALDH2∗1/∗2* allele carriers. Therefore, *ALDH2∗2/∗2* allele carriers cannot consume alcohol.^18^ This was consistent with this present study. In addition, this phenomenon might help to reduce developing AF. The Central Illustration shows the relationship between *ALDH2* genotypes and AF development. As shown, although small amounts of alcohol did not lead to AF development in *ALDH2* wild-type carriers, large amounts of alcohol led to AF development despite, normal *ALDH2* metabolism. However, even small amounts of alcohol led to development of AF in *ALDH2*-deficient variant carriers because of low *ALDH2* metabolism activity.

**Central Illustration:**
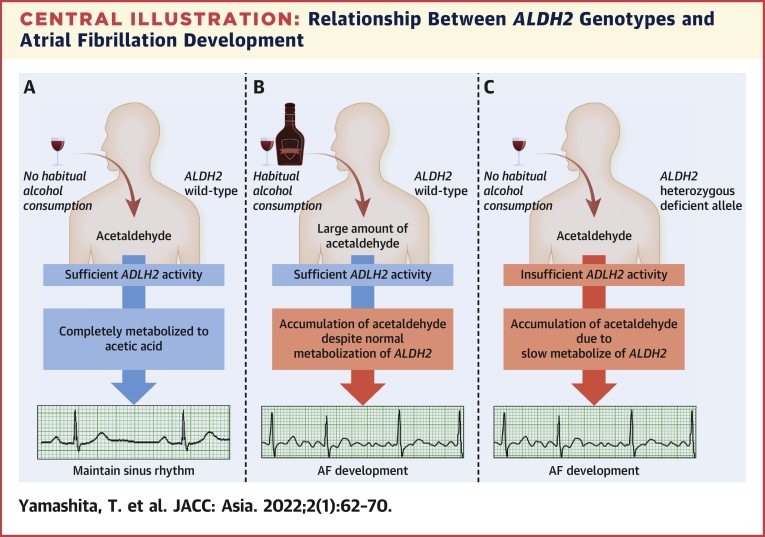
Relationship Between *ALDH2* Genotypes and Atrial Fibrillation Development **(A)** Consumption of a normal amount of alcohol in *ALDH2* wild-type. **(B)** Consumption of a large amount of alcohol in *ALDH2* wild-type. **(C)** Consumption of a normal amount of alcohol in *ALDH2*-deficient variant. AF = atrial fibrillation.

Therefore, it might be important to identify whether an individual is an *ALDH2∗1/∗2* allele carrier, because both *ALDH2* wild-type and *ALDH2∗1/∗2* allele carriers can consume alcohol. Symptoms of alcohol flushing syndrome could be a predictor to differentiate between *ALDH2* wild-type and *ALDH2∗1/∗2* allele carriers, because in the *ALDH2∗1/∗2* allele carriers, facial flushing, headache, nausea, and palpitations (the typical symptoms of alcohol flushing syndrome) have been observed even with small amount of alcohol.^17^

### STUDY LIMITATIONS

This study’s population only included patients who were admitted to the Cardiovascular Center of Kumamoto University. In addition, the 385 patients of 656 patients underwent catheter ablation for AF. Although the proportion of the *ALDH2* genotype was consistent with that in a previous report, it might not represent the general population.

Although the relationship between AF and sleep apnea syndrome was not evaluated, obesity was not a risk factor of AF in this study. This might be because most patients with the sleep apnea syndrome in the Japanese population develop the condition from having a small jaw and not because of obesity.^22^^,^^23^

Socioeconomics factors, educational attainment, and regional customs demonstrated a close relationship with habitual alcohol consumption.^24^^,^^25^ Although we could not gather enough information related to these data, they might be important predictors.

Although a correlation between *ALDH2* genotypes and new-onset AF was not observed, the follow-up period was short because of retrospective study design. Increasing the follow-up period and patient numbers might clarify this correlation.

The prevalence of *ALDH2∗2* allele carriers is extremely rare in Europeans^14^; therefore, the same result might not be obtained in a European cohort.

The duration from beginning habitual alcohol consumption to developing AF might be different for each *ALDH2* genotype. However, the exact starting time of habitual alcohol consumption, especially the starting time of the current drinking volume, was obscure. In addition, some patients were asymptomatic for AF. Therefore, this evaluation could not be conducted.

## Conclusions

Although the *ALDH2∗1/∗2* itself was not associated with AF, *ALDH2∗1/∗2* carriers with habitual alcohol consumption might experience AF because of slow alcohol metabolism. In contrast, *ALDH2∗2/∗2* carriers had a lower incidence of AF. This might be related to the absence of habitual alcohol consumption in *ALDH2∗2/∗2* carriers because of the negligible activity of *ALDH2.* Thus, abstaining from alcohol consumption could prevent the development of AF in patients who are *ALDH2∗1/∗2* allele carriers in the Japanese population.

Perspectives **COMPETENCY IN MEDICAL KNOWLEDGE:** Alcohol—a risk factor for AF—is metabolized by *ALDH2*. Dysfunctional alleles of the *ALDH2* (*ALDH2*-deficient variants) are prevalent among East Asians. In this study, we revealed that habitual alcohol consumption in *ALDH2*-deficient variant carriers is an independent risk factor for AF because of the presence of slow alcohol metabolism.

**TRANSLATIONAL OUTLOOK:** It should be noted that habitual alcohol consumption in *ALDH2*-deficient variant carriers is an independent risk factor for AF because of the presence of slow alcohol metabolism. Therefore, abstaining from alcohol consumption, particularly by *ALDH2*-deficient variant carriers, may suppress AF onset.

## Funding Support and Author Disclosures

This work was supported by JSPS KAKENHI Grant Number JP20K22878. Drs Hoshiyama and Kanazawa have received grants from Medtronic Japan, Nihon Kohden, Abbott Medical Japan, Fukuda Denshi, Boston Scientific Japan, Japan Lifeline, Nipro, and Biotronik Japan. Dr Tsujita has received honoraria from Bayer Yakuhin, Daiichi-Sankyo, Kowa, MSD, Sanofi, and Takeda Pharmaceutical; and has received grants from Astellas Pharma, Abbott Vascular Japan, Bayer Yakuhin, Boehringer Ingelheim Japan, Boston Scientific Japan, Bristol Myers, Chugai Pharmaceutical, Daiichi-Sankyo, Goodman, Japan Lifeline, Medtronic Japan, Mitsubishi Tanabe Pharma, MSD, Novartis Pharma, Otsuka Pharmaceutical, Sanofi, Takeda Pharmaceutical, and Terumo. All other authors have reported that they have no relationships relevant to the contents of this paper to disclose.
