# Supplementary material for: Effect of the ALDH2 Variant on the Prevalence of Atrial Fibrillation in Habitual Drinkers
Source: JACC Asia. 2022 Jan 18;2(1):62–70. doi: 10.1016/j.jacasi.2021.10.009 (PMC9627901; doi:10.1016/j.jacasi.2021.10.009)
Supplement: Supplemental Data [file mmc1.docx]

**Effect of the *ALDH2* Variant on the Prevalence of Atrial Fibrillation in Habitual Drinkers**

**Brief Title: ALDH2 variants affect AF in habitual drinkers**

Takayoshi Yamashita, MD, PhD^a^, Yuichiro Arima, MD, PhD^a^, Tadashi Hoshiyama, MD, PhD^a^, Noriaki Tabata, MD, PhD^a^, Daisuke Sueta, MD, PhD^a^, Yusei Kawahara, MD^a^, Miwa Ito, MD, PhD^a^, Hisanori Kanazawa, MD, PhD^a^, Masanobu Ishii, M.D., PhD^a^, Kenshi Yamanaga MD. PhD^a^, Shinsuke Hanatani, MD, PhD^a^, Seiji Takashio, MD, PhD^a^, Satoshi Araki, MD, PhD^a^, Satoru Suzuki, MD, PhD^a^, Eiichiro Yamamoto. MD, PhD^a^, Koichi Kaikita, MD, PhD^a^, Kentaro Oniki, PhD^b^, Junji Saruwatari, PhD^b^, Kenichi Matsushita, MD, PhD^a^ and Kenichi Tsujita, MD, PhD^a^

In the present study, LADs, proportion of AF, and drinking habit between *ALDH2* genotypes were significantly different. which might lead to bias in this analysis. Therefore, propensity-score matching analysis was performed between *ALDH2* wild-type and *ALDH2*1/*2* carriers after excluding *ALDH2*2/*2* genotypes due their small number. The proportion between *ALDH2* wild-type and *ALDH2*1/*2* carriers was almost 2:1; therefore, propensity-score matching analysis was performed at 2:1. As a result, this analysis matched 450 patients; *ALDH2* wild-type (n = 300) and *ALDH2*1/*2* carriers (n = 150).

Supplemental Table 1 shows the patients characteristics following propensity-score matching analysis. As shown in this table, all backgrounds have no significant difference.

Supplemental Table 2 shows the relationship between AF and variables among these patients. Although habitual alcohol consumption indicates a high odds ratio, *ALDH2* variant itself is not a risk factor of AF. However, as shown int the Supplemental Table 3, *ALDH2* wild-type carriers with habitual alcohol consumption (OR: 2.14, P = 0.04), and *ALDH2*1/*2* allele carriers with habitual alcohol consumption (OR: 5.96, P = 0.001) positively correlated with the risk of AF. Particularly, *ALDH2*1/*2* allele carriers who consumed alcohol habitually showed the highest OR among these variables. The numbers changed following propensity-score matching, but the meaning and significant difference did not change.

Supplemental Table 4 shows the relationship between habitual alcohol consumption and AF prevalence among *ALDH2* genotypes using inversed probability treatment weighting analysis. This table shows that the *ALDH2* variant itself is not a risk factor for AF (OR:1.12, P = 0.57). However, *ALDH2* wild-type (OR: 2.40, P < 0.001), and *ALDH2*1/*2* allele (OR: 4.47, P = 0.001)　carriers with habitual alcohol consumption positively correlated with the risk of AF development. Particularly, *ALDH2*1/*2* allele carriers who consumed alcohol habitually showed the highest OR among these variables.

Supplemental Table 1. Patient’s characteristics

|  | all n:450 | *ALDH2* wild n:300 | *ALDH2*1/*2* n:150 | p value |
| --- | --- | --- | --- | --- |
| age | 64 (54–72) | 64 (53–71) | 64 (56–71) | 0.84 |
| male: n (%) | 254 (56.4) | 168 (56.0) | 86 (57.3) | 0.84 |
| BMI (kg/m^2^) | 23.2 (21.0–25.6) | 23.2 (21.1–25.7) | 23.3 (20.8–25.4) | 0.64 |
| BNP (pg/mL) | 29.9 (13.3–69.1) | 30.7 (13.8–66.8) | 27.1 (12.0–69.8) | 0.71 |
| EF (%) | 63.3 (59.7–66.2) | 63.6 (59.9–66.7) | 62.9 (59.6–65.5) | 0.08 |
| LADs (mm) | 36.0±6.4 | 36.4±6.4 | 35.3±6.3 | 0.09 |
| eGFR (mL/min/1.73m2) | 72.0 (63.0–85.0) | 73.0 (63.8–85.0) | 72.0 (61.3–84.8) | 0.39 |
| atrial fibrillation: n (%) | 279 (62.0) | 186 (62.0) | 93 (62.0) | 1.00 |
| hypertension: n (%) | 234 (52.0) | 161 (53.7) | 73 (48.7) | 0.65 |
| diabetes mellitus: n (%) | 55 (12.2) | 35 (11.7) | 20 (13.3) | 0.65 |
| drinking habit: n (%) | 153 (34.0) | 102 (34.0) | 51 (34.0) | 1.00 |
| current smoking: n (%) | 78 (17.3) | 53 (17.7) | 25 (16.7) | 0.90 |

* *ALDH2* = aldehyde dehydrogenase, EF = ejection fraction, LADs = left atrial dimension

Supplemental Table 2. Relationship between atrial fibrillation and variables

|  | Univariate analysis | | |  | Multivariate analysis | | |
| --- | --- | --- | --- | --- | --- | --- | --- |
| **variables** | **OR** | **95%CI** | **p value** |  | **OR** | **95%CI** | **p value** |
| age over 60 | 2.63 | 1.76 – 3.92 | <0.001 |  | 2.66 | 1.65 – 4.29 | <0.001 |
| Hypertension | 2.71 | 1.83 – 4.02 | <0.001 |  | 1.92 | 1.23 – 3.01 | 0.04 |
| obesity | 1.19 | 0.78 – 1.80 | 0.42 |  | 0.99 | 0.61 – 1.60 | 0.97 |
| Diabetes mellitus | 1.30 | 0.71 – 2.36 | 0.39 |  | 0.93 | 0.48 – 1.80 | 0.84 |
| male | 2.79 | 1.89 – 4.14 | <0.001 |  | 2.21 | 1.37 – 3.55 | 0.001 |
| alcohol | 4.39 | 2.73 – 7.05 | <0.001 |  | 3.02 | 1.78 – 5.16 | <0.001 |
| *ALDH2*1/*2* | 1.00 | 0.67 – 1.50 | 1.00 |  | 1.03 | 0.66 – 1.61 | 0.90 |

**ALDH2* = aldehyde dehydrogenase

Supplemental Table 3. Relationship between atrial fibrillation and variables

| **variables** | | **OR** | **95%CI** | **p value** |
| --- | --- | --- | --- | --- |
| age over 60 | | 2.81 | 1.74 – 4.55 | <0.001 |
| Hypertension | | 1.87 | 1.20 – 2.93 | 0.006 |
| obesity | | 1.01 | 0.63 – 1.64 | 0.97 |
| Diabetes mellitus | | 0.87 | 0.45 – 1.67 | 0.67 |
| male | | 2.22 | 1.38 – 3.58 | 0.001 |
| *ALDH2* genotype | habitual alcohol consumption |  |  |  |
| *ALDH2* wild | no |  | reference |  |
| *ALDH2* wild | yes | 2.59 | 1.40 – 4.81 | 0.02 |
| *ALDH2*1/*2* | no | 0.98 | 0.59 – 1.63 | 0.93 |
| *ALDH2*1/*2* | yes | 4.07 | 1.64 – 10.1 | 0.003 |

**ALDH2* = aldehyde dehydrogenase

Supplemental Table 4. Relationship between atrial fibrillation and variables using inversed probability weighting analysis

| **variables** | | **OR** | **95%CI** | **p value** |
| --- | --- | --- | --- | --- |
| *ALDH2*1/*2* | | 1.12 | 0.76 - 1.65 | 0.57 |
| *ALDH2* genotype | habitual alcohol consumption |  |  |  |
| *ALDH2* wild | no |  | reference |  |
| *ALDH2* wild | yes | 2.40 | 1.60 - 3.60 | <0.001 |
| *ALDH2*1/*2* | no | 1.14 | 0.73 - 1.77 | 0.58 |
| *ALDH2*1/*2* | yes | 4.47 | 1.85 - 10.84 | 0.001 |

Supplemental Table 5. Relationship between new atrial fibrillation onset and variables in follow up patients

| **variables** | **OR** | **95%CI** | **p value** |
| --- | --- | --- | --- |
| age over 60 | 3.90 | 0.47 - 32.42 | 0.20 |
| Hypertension | 0.10 | 0.01 - 1.26 | 0.08 |
| obesity | 0.94 | 0.08 - 10.64 | 0.96 |
| male | 4.43 | 0.42 - 47.22 | 0.23 |
| habitual alcohol consumption | 1.46 | 0.17 - 12.89 | 0.73 |
| *ALDH2* genotype |  |  |  |
| *ALDH2* wild |  | reference |  |
| *ALDH2*1/*2* | 1.52 | 0.19 - 12.16 | 0.69 |
| *ALDH2*2/*2* | 6.89 | 0.59 - 80.01 | 0.12 |

**ALDH2* = aldehyde dehydrogenase

Supplemental Table 6. Relationship between new atrial fibrillation onset and variables in follow up patients

| **variables** | | **OR** | **95%CI** | **p value** |
| --- | --- | --- | --- | --- |
| age over 60 | | 3.03 | 0.46 - 19.76 | 0.25 |
| Hypertension | | 0.16 | 0.15 - 1.66 | 0.12 |
| obesity | | 0.69 | 0.07 - 7.10 | 0.75 |
| male | | 4.52 | 0.47 - 43.89 | 0.19 |
| *ALDH2* genotype | habitual alcohol consumption |  |  |  |
| *ALDH2* wild | no |  | reference |  |
| *ALDH2* wild | yes | 1.36 | 0.08 - 24.42 | 0.84 |
| *ALDH2*1/*2* | no | 1.16 | 0.07 - 19.29 | 0.92 |
| *ALDH2*1/*2* | yes | 4.28 | 0.25 - 74.42 | 0.32 |
| *ALDH2*2/*2* | no | 10.56 | 0.88 – 127.00 | 0.06 |

**ALDH2* = aldehyde dehydrogenase
